# User Perceptions of Behavioral Change Strategies in Diabetes Apps: Feedback From Online Support Groups

**DOI:** 10.1177/19322968251343918

**Published:** 2025-05-24

**Authors:** Eirik Årsand, Elia Gabarron, Pietro Randine

**Affiliations:** 1 Department of Computer Science, Faculty of Science and Technology, UiT The Arctic University of Norway, Tromsø, Norway; 2 Department of Education, ICT and Learning, Østfold University College, Halden, Norway

**Keywords:** diabetes mellitus, mobile applications, behavior and behavior mechanisms, patient preference, health behavior

## Abstract

**Background:**

Behavioral change strategies are used in mobile health applications to help individuals manage chronic conditions like diabetes. However, there is limited research on user preferences and perceptions regarding these strategies in the context of diabetes management apps. This study aimed to investigate the preferences of individuals with diabetes and their relatives concerning behavioral intervention functions used in mobile health apps to enhance the design and effectiveness of future applications.

**Methods:**

An online survey was conducted to gather sociodemographic information, details about diabetes diagnoses, and the target group’s preferences for the use of nine main behavioral change strategies, possible to include in mobile health apps. Participants were asked to rate their agreement with specific statements related to each of the nine strategies on a three-point scale: “Agree,” “Don’t know,” or “Disagree.” Recruitment efforts targeted 12 diabetes support groups on Facebook.

**Results:**

A total of 107 responses were received, all from Norwegian Facebook groups. The most valued behavior intervention function for diabetes apps was enablement, where 85% of the respondents wanted app functions based on this. Second, environmental restructuring received 70.1% votes, followed by incentivization and training, with 68.2% and 67.3%, respectively.

**Conclusions:**

We identified that the users in this survey preferred more, and other behavior change strategies that were identified were used in a recent review. We conclude that more awareness is needed among app developers of preferences among end users.

## Introduction

Applications for mobile phones (apps) are increasingly getting more used and more effective in disease self-management, including diabetes self-management.^1-4^ However, there is significant potential to integrate health-related sensors,^5^ digital platforms, and programmable algorithms into these smartphone apps to improve disease management further.^6^ The most valuable applications are often those developed in collaboration with end users, specifically individuals living with diabetes, highlighting the importance of user-centered design.^7^ Gathering feedback and preferences from end users can be done in many ways, eg, arranging focus group meetings, interviews, and feasibility studies—including surveys among target groups on social media, which has shown to be a quick and useful method.^6,8^

**Figure 1.**
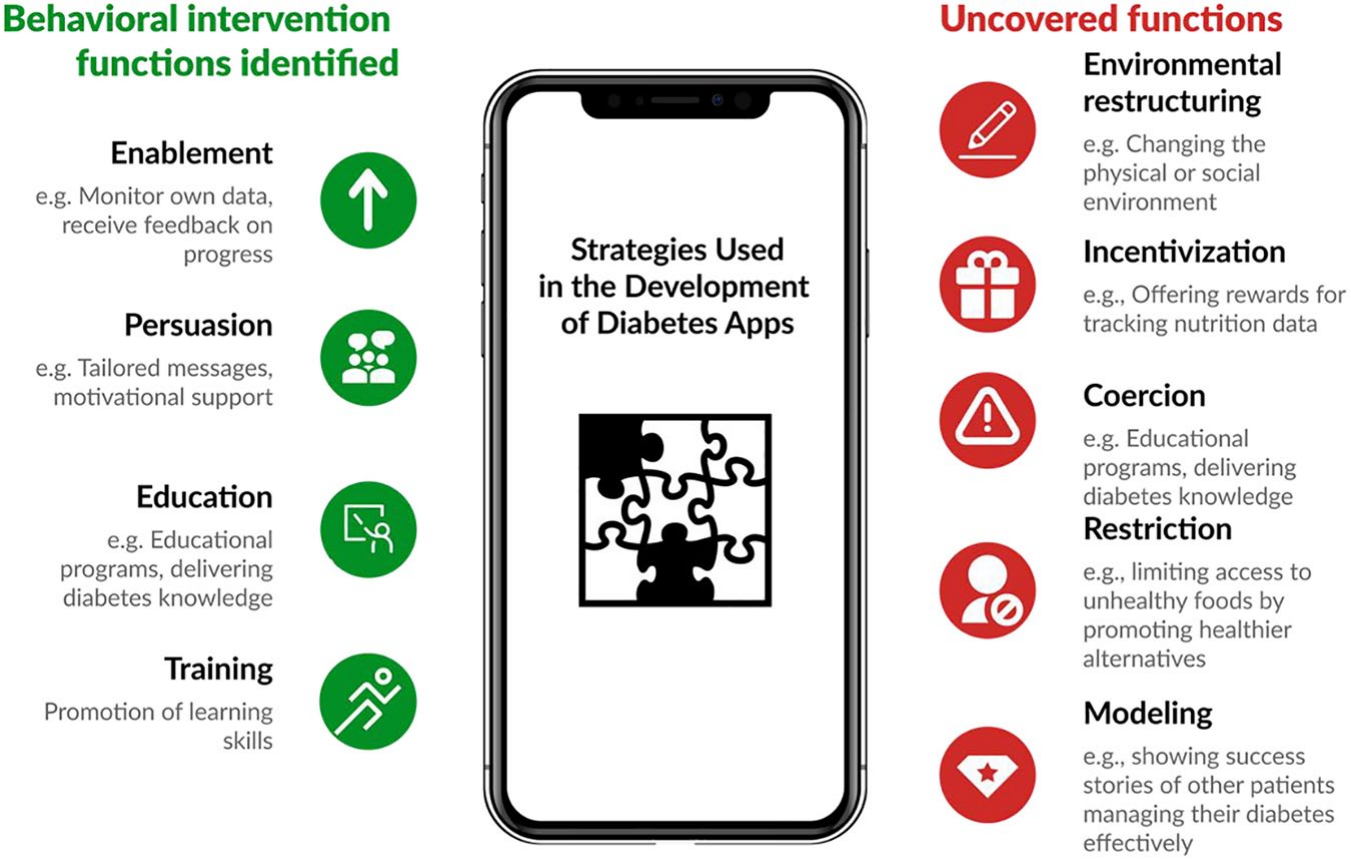
Identified vs uncovered behavioral intervention functions: a visual comparison.^11^

To improve health-related apps, evidence-based behavioral frameworks can guide their development. The Behavior Change Wheel (BCW) and its framework describing how Capability, Opportunity, and Motivation lead to Behavior (COM-B)^9^ are being applied in interventions addressing diabetes, both digital and non-digital, to enhance health behaviors and overcome barriers.^10^ This framework provides a structured approach to encourage diabetes self-management through nine intervention functions: education; persuasion; incentivization; coercion; training; restriction; environmental restructuring; modeling; and enablement (see Figure 1 for examples of use of behavior intervention functions).

In the context of non-digital interventions, this framework has been used in several studies, including the design of a randomized controlled trial (RCT) involving dietary interventions for women with gestational diabetes mellitus (GDM), focusing on the use of persuasion, education, environmental restructuring, training, incentivization, enablement, and modeling.^12^ A study analyzing the impact of a dietary intervention using the BCW framework identified that providing social support, social comparison, and credible information influenced significantly positive health behaviors among individuals with type 2 diabetes (T2D).^13^ In addition, techniques like self-monitoring, action planning, and behavioral regulation were found crucial for increasing self-efficacy and habit formation.^13^ Moreover, a framework developed to address barriers to insulin treatment identified that key intervention functions were education, persuasion, modeling, enablement, training, and coercion. These were employed for varied purposes, including shaping beliefs and improving self-efficacy.^14^ Similarly, a pilot dietary and exercise intervention for T2D, which leveraged education, persuasion, incentivization, and enablement, demonstrated significant improvements in the intervention group, highlighting the effectiveness of these behavior change techniques.^15^

In digital interventions, the BCW and COM-B frameworks have similarly guided the development of effective solutions. A study on an information technology (IT)-enabled health coaching and resource linkage program for low-income Latina mothers with GDM highlighted that persuasion and training were key for addressing beliefs about consequences of behaviors and improving self-efficacy. The inclusion of narratives with emotional content resonated with participants, reinforcing behavior change.^16^ Another example was a study on an app designed to increase physical activity in women with GDM.^17^ This app incorporated various strategies, including education, training, enablement, environmental restructuring, and persuasion, showcasing the comprehensive utility of these intervention functions. Furthermore, a literature review of apps targeting T2D revealed that enablement, persuasion, education, and training were the most commonly employed intervention functions, reflecting their centrality in digital health behavior change strategies.^11^ However, other BCW functions—such as incentivization, coercion, restriction, environmental restructuring, and modeling—were notably absent from the analyzed studies in this review, as summarized in Figure 1.

Although the BCW framework is widely used in diabetes interventions, there is limited understanding of which intervention functions are most valued by diabetes app users. Understanding these preferences is essential for designing effective and user-centered interventions, as it can help tailor app features to better align with users’ needs and enhance engagement and self-management. To our knowledge, no research has yet assessed the preferences of diabetes app users regarding the behavior change intervention functions as outlined by the BCW framework.^9^

### Aim of the Study

The objective of this study is to evaluate diabetes app users’ perceptions for behavior intervention functions that are applied or could be applied to the apps they use or want to use.

## Methods

The Norwegian agency Shared Services in Education and Research (Sikt) approved the study and recruitment method (project reference number 758884). The recruitment was done between December 2024 and January 2025.

### Measures

We created a questionnaire using Nettskjema, a tool for designing and conducting online questionnaires, to collect data on various metrics anonymously on a secure platform. The questionnaire included questions regarding sociodemographic information, including their age group, sex, diabetes type, and participants’ opinions on the nine behavioral change strategies in the BCW, exemplified by examples relevant to diabetes management. These examples were descriptions of intervention functions in a health app, with screenshots of a diabetes-related functionality. Participants were asked to indicate their level of agreement with statements related to each strategy on a three-point scale: “Agree,” “Don’t know,” or “Disagree.” Choosing only three options for these agreements was chosen since the BCW includes relatively complex terminology; thus, we thought the users should be asked to give simple feedback such as these three options. The questionnaire was initially developed in English, as outlined in Supplemental Appendix 1, and was subsequently translated into Norwegian, available in Supplemental Appendix 2.

### Sample and Procedure

The initial selected representative social media groups were three English-language diabetes support groups on Facebook, having 34k members, 101k members, and 54k members. A text with a link to the survey was submitted, but none of these support groups agreed to post the survey in their group. After one week, six new support groups were asked to publish the recruitment post, ie, groups of 3.4k members, 155k members, 13k members, 26k members, 40k members, and 26k members, with the same results of no positive feedback for posting in their Facebook groups. The list of groups is available upon request from the corresponding author. In January 2025, we asked three Norwegian diabetes support groups on Facebook, with 11k members, 4.6k members, and 152 members. Two groups responded positively within a few hours, and one within a few days.

Participants were recruited through a multi-step process. First, the first author requested membership in the diabetes support groups. After receiving approval from the group moderators, the moderators of these groups were contacted with a formal request to post the survey invitation. Messages were sent to explain the purpose of the study, assuring participants that the questionnaire would be brief (taking only 2-3 minutes), emphasizing that no personal information would be collected, and promising to share the results after the study was completed.

The sample size was calculated using a standard formula for finite populations, considering the number of members in 12 representative social media groups focused on diabetes. A 95% confidence level, a 10% margin of error, and a 50% variability were considered. This calculation resulted in a requirement of a sample of 96 participants, considered suitable for reliably representing the preferences of diabetes app users.

### Analysis

The data set was exported from Nettskjema, with labels mapped using a codebook to convert encoded values into descriptive categories (eg, age ranges, gender, and responses like “Agree”). Visualizations were generated using Matplotlib and edited with Figma.

## Results

A total of 107 individuals responded to the survey, all recruited from Norwegian social media support groups. Seventy-two answers were received within the first five days, while the remaining were collected over the following seven days. *The average response time for completing the survey was approximately* 4 minutes and 9 seconds *(SD ≈ 4 minutes).*

### Age Range Distribution by Sex and Participant Characteristics

**Figure 2.**
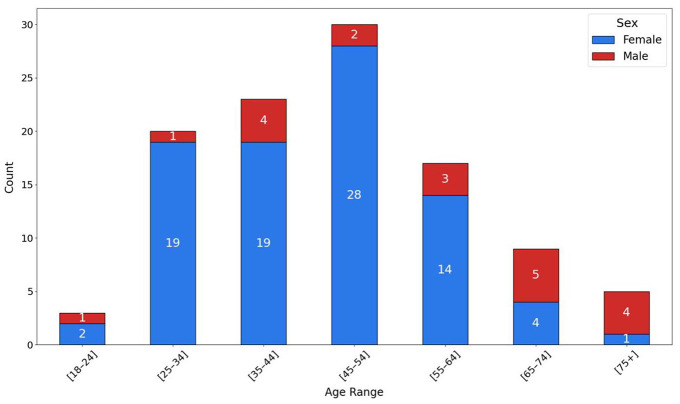
Age range distribution by sex and participant characteristics.

The chart in Figure 2 illustrates the age range distribution of questionnaire respondents by sex.

Most participants (81.3%) identified as female, while 18.7% identified as male. The distribution shows a higher concentration of respondents in the age groups 35 to 44 and 45 to 54, with the latter being the largest group (30 respondents). Age groups 55 to 64 and 25 to 34 followed, while the youngest (18-24) and oldest (75+) age brackets were underrepresented.

In terms of diabetes type, most respondents (72 individuals) reported having type 1 diabetes (T1D), while 31 participants identified as having T2D. An additional l4 respondents reported not having diabetes themselves but being closely related to someone with diabetes. These responses included “Mother of a child with Type 1 diabetes,” “I do not have diabetes, but my child does,” “Diagnosed as both Type 2 and Type 1,” “Mixed diabetes,” “Relative of a child with diabetes,” and “Maturity-Onset Diabetes of the Young.”

### User Perceptions for Behavioral Intervention Functions

**Figure 3.**
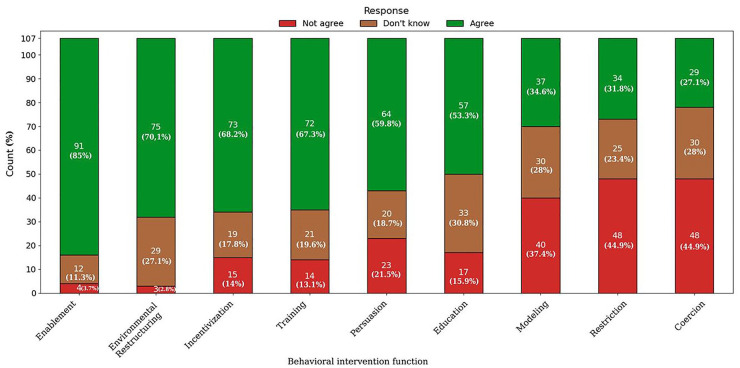
User preferences for behavioral intervention functions.

Figure 3 illustrates user perceptions for the nine behavioral intervention functions based on the BCW. Participants were asked to express their agreement with statements representing each function, with responses categorized as “Agree,” “Don’t know,” or “Not agree.”

Enablement emerged as the most strongly supported function, with 85% of respondents in favor of its use in diabetes apps. Only 11.3% were unsure, while a minimal 3.7% disagreed. Environmental restructuring also received high praise, with 70.1% agreement among the respondents.

Also, incentivization and training gained high scores, with 68.2% and 67.3%, respectively. Persuasion received moderate support, with a 59.8% agreement score. Education was endorsed by 53.3% of the respondents; however, a notable 30.8% expressed uncertainty and 15.9% disagreement regarding app functions based on education.

Modeling received mixed feedback, with only 34.6% in favor of such functions. Restriction was even less favored, attracting only 31.8% agreement, while a significant 44.9% expressed opposition, indicating that restrictive strategies may not align with user preferences for apps’ functionalities. Finally, coercion was the least valued function, with only 27.1% agreement, and the majority, at 44.9%, opposed its usage in apps, reflecting resistance to penalties or reminders for non-compliance.

### Comments to the Social Media Recruitment Posts

From some of the readers of the recruitment post, we received these feedback messages about diabetes apps:

I miss an alternative for my app to talk with my insulin pump so that injections and blood glucose graphs automatically are updated.

Those apps that exists, I cannot use neither on my Redmi (Xiaomi brand) or Huawey phone.

Related to food advice—these are for me not useful if I cannot add preferences, e.g. that I can exclude food I don’t like or is allergic to.

## Discussion

### Summary of Findings

We recruited 107 respondents, all from Norwegian Facebook groups, where 81.3% identifying as female and 18.7% as male. Most participants, 30 (28%), were aged 45 to 54, 72 (67.3%) had T1D, 31 (29%) had T2D, and 4 (3.7%) were relatives of someone with diabetes. On average, the survey took 4 minutes and 9 seconds to complete.

The most valued behavior intervention function for diabetes apps was enablement, which received an 85% agreement for its inclusion. Following this, environmental restructuring received 70.1% agreement, while incentivization and training received 68.2% and 67.3%, respectively. Persuasion (59.8%) and education (53.3%) received moderate support, while modeling (34.6%), restriction (31.8%), and coercion (27.1%) were the least preferred.

### Behavioral Intervention Functions—Literature vs Survey

A previous review^11^ identified that enablement, persuasion, education, and training were the most commonly reported functions integrated into diabetes apps. Notably, other functions such as incentivization, coercion, restriction, environmental restructuring, and modeling were entirely absent.

The survey findings offer a different perspective by capturing the users’ perceptions regarding these functions. More than 70% agreed that enablement and environmental restructuring were valuable in diabetes apps. This discrepancy indicates that individuals with T1D, who comprised the majority of respondents in the survey, may be seeking external factors and environmental changes to support their self-management. Examples of these factors include better access to healthy food options and improved access to glucose monitoring devices.^10^ This aligns with findings from another study,^18^ which discusses the role of environmental restructuring in promoting healthier behaviors for diabetes self-management. These findings highlight a significant opportunity for future diabetes app development. By incorporating environmental restructuring features, apps could more effectively meet user needs. Implementing such elements could increase user engagement and enhance self-management outcomes, potentially reducing user dropout rates.^19^

The users in the survey are in line with the literature findings for valuing functions including training with a preference of 67.3%, and moderately also supporting education. Despite being featured in eight studies from the literature review, education received only 53.3% agreement in the survey.

Moreover, while we, in the review, found that incentivization, restriction, and modeling were not reported in articles about diabetes apps, the survey indicated moderate support for these intervention functions. For instance, incentivization received 68.2% agreement, suggesting that users see value in rewards or positive reinforcement for meeting goals. Research on digital health interventions also supports the effectiveness of incentives, such as financial rewards,^20^ but it is also challenging to define successful incentives.^21^ Modeling got only 34.6% agreement, reflecting limited interest in learning from success stories or role models. Restriction was less popular at 31.8% agreement but still got support from some respondents, which contrasts with its absence in existing apps. Both the literature review and the survey consistently identified that coercion is considered as a less desirable approach. The survey indicated that 44.9% of users disagreed with diabetes app functions based on this strategy. The relatively large but stable percentage of “Don’t know” responses could indicate that between 20% and 30% of the users did find the nine questions related to BC intervention functions too hard to answer, thus selected this option.

These findings suggest that future diabetes apps could consider incorporating environmental restructuring and incentivization functionalities to improve user engagement and long-term usage. Addressing these user preferences in app development may enhance the effectiveness of self-management and overall user satisfaction. Ideally, the future apps should be possible to tailor to the user preferences, eg, in the start-up phase of using the app. Some could be manually adjusted and other might be automatically adjusted based on the actual use of the app. Even though there are many diabetes apps available, we see few that have many features and behavior intervention functions.

### Recruiting Survey Participants via Social Media

Recruiting survey participants through social media is a common practice that has grown in health research in recent years.^22^ However, the literature indicates that this recruitment method can be challenging due to several factors ^23,24^. Many users may perceive online surveys as time-consuming, irrelevant to their interests, or they may feel survey fatigue, as they are continuously asked to fill surveys^25^ leading to low engagement. The overwhelming amount of content on social media can lead to survey invitations being overlooked or ignored.

Trust issues and privacy concerns may also discourage participation, as some users can doubt the legitimacy of surveys posted online and may be cautious about sharing personal information on platforms known for data misuse. Using anonymous surveys and directing interested participants through a survey link to a landing page for study information are suggested as mitigation strategies.^26,27^ Avoiding long surveys and providing information about the study purpose are recommended to improve response rates.^25^ Surveys offering monetary and nonmonetary incentives have proven to be more effective than recruitment offering no incentive.^28^ Increased response rates in individual studies have also been observed with studies that used pre-contact via a peer’s phone call, personalized messages, messages sent on Fridays, and using registered e-mails.^28,29^

In this study, we aimed to recruit social media users from representative Facebook groups focused on diabetes, specifically targeting English-speaking groups and Norwegian-speaking groups. However, we were only able to recruit from Norwegian-speaking social media groups. This may reflect stronger engagement or trust in national researchers, highlighting the challenges of recruiting a diverse, international participant pool for studies like this. Future research could explore the effectiveness of various strategies to increase response rates to online surveys, especially for recruiting diverse and international participants. Notable is also that the recruitment post generated useful feedback as well, emphasizing the importance of explaining the purpose of the study well when recruiting.

### Limitations

Our study has several limitations. Although the questionnaire underwent multiple rounds of internal testing and refinement among the authors, it was not formally piloted or psychometrically validated, making it uncertain how consistently participants interpreted the intended meaning of each item.^30^ In addition, the survey measured participants’ agreement with specific examples illustrating each BCW intervention function rather than generalized preferences for the functions themselves. Thus, the results should be interpreted as indicative rather than conclusive for all nine BCW functions. Some formulations may overlap with constructs from other behavioral frameworks, which could introduce variability in interpretation. Framing effects may also have influenced participants’ responses, particularly for negatively connoted terms like “Coercion.” Although we adhered to BCW terminology, negative labeling may have biased participants’ ratings, as shown in decision-making and consumer behavior research,^31,32^ and likely relevant for health survey contexts.

Another limitation is that all participants were recruited from Norwegian Facebook groups, which may limit the generalizability of the findings to individuals who use other social media platforms or who are not active on social media. Although the sample size (n = 107) exceeded the minimum required for statistical validity, it cannot be considered representative of the broader population of diabetes app users. Recruitment challenges, including group administrators declining to share the survey link, further reduced the number of participants.

Using an online survey may also have excluded individuals less comfortable with digital platforms. The three-point response scale (“Agree,” “Don’t know,” “Disagree”) likely limited the depth of participants’ feedback. Although anonymity may have encouraged honesty, it could also have reduced the level of thoughtful engagement. Response times varied substantially (mean: 4 minutes and 9 seconds; SD: ~4 minutes), suggesting differences in participant attention.

## Conclusions

The findings highlight user perceptions for behavioral functions in diabetes apps, with a particular preference for enablement and environmental restructuring. Participants showed moderate support for strategies like training and incentivization, while they expressed the least preference for coercion and restriction, indicating a general resistance to punitive approaches.

Most respondents reported having T1D, which means the findings may not fully reflect the preferences of individuals with T2D. Our findings provide insight into what diabetes app users—primarily those with T1D—value in behavior change strategies.

Future diabetes app development should prioritize integrating the identified user-preferred behavior change features, and research should be made to see whether these will improve the usefulness and effect of diabetes apps.

## Supplemental Material

sj-docx-1-dst-10.1177_19322968251343918 – Supplemental material for User Perceptions of Behavioral Change Strategies in Diabetes Apps: Feedback From Online Support Groups Supplemental material, sj-docx-1-dst-10.1177_19322968251343918 for User Perceptions of Behavioral Change Strategies in Diabetes Apps: Feedback From Online Support Groups by Eirik Årsand, Elia Gabarron and Pietro Randine in Journal of Diabetes Science and Technology

sj-docx-2-dst-10.1177_19322968251343918 – Supplemental material for User Perceptions of Behavioral Change Strategies in Diabetes Apps: Feedback From Online Support Groups Supplemental material, sj-docx-2-dst-10.1177_19322968251343918 for User Perceptions of Behavioral Change Strategies in Diabetes Apps: Feedback From Online Support Groups by Eirik Årsand, Elia Gabarron and Pietro Randine in Journal of Diabetes Science and Technology
