# Supplementary material for: User Perceptions of Behavioral Change Strategies in Diabetes Apps: Feedback From Online Support Groups
Source: J Diabetes Sci Technol. 2025 May 24;19(5):1239–46. doi: 10.1177/19322968251343918 (PMC12104217; doi:10.1177/19322968251343918)
Supplement: sj-docx-1-dst-10.1177_19322968251343918 – Supplemental material for User Perceptions of Behavioral Change Strategies in Diabetes Apps: Feedback From Online Support Groups [file sj-docx-1-dst-10.1177_19322968251343918.docx]

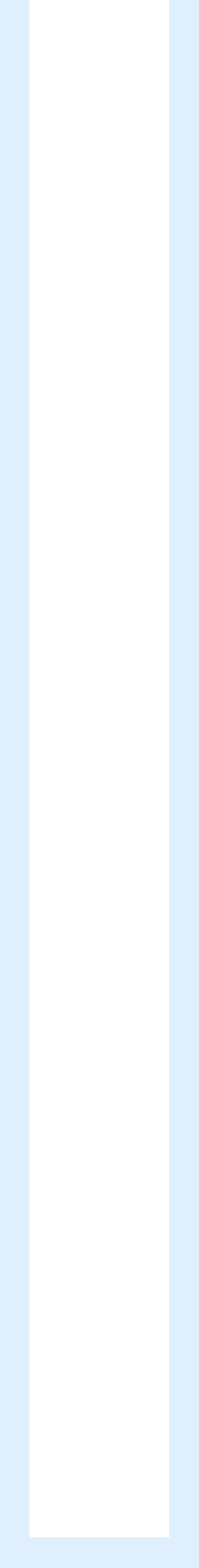

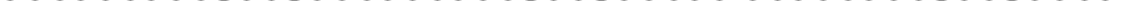

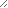

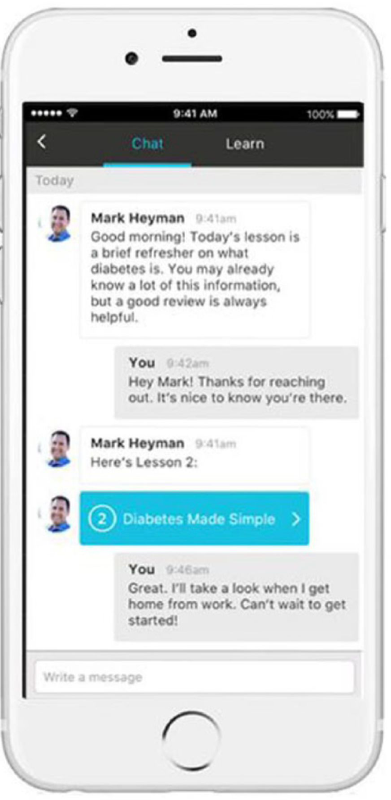

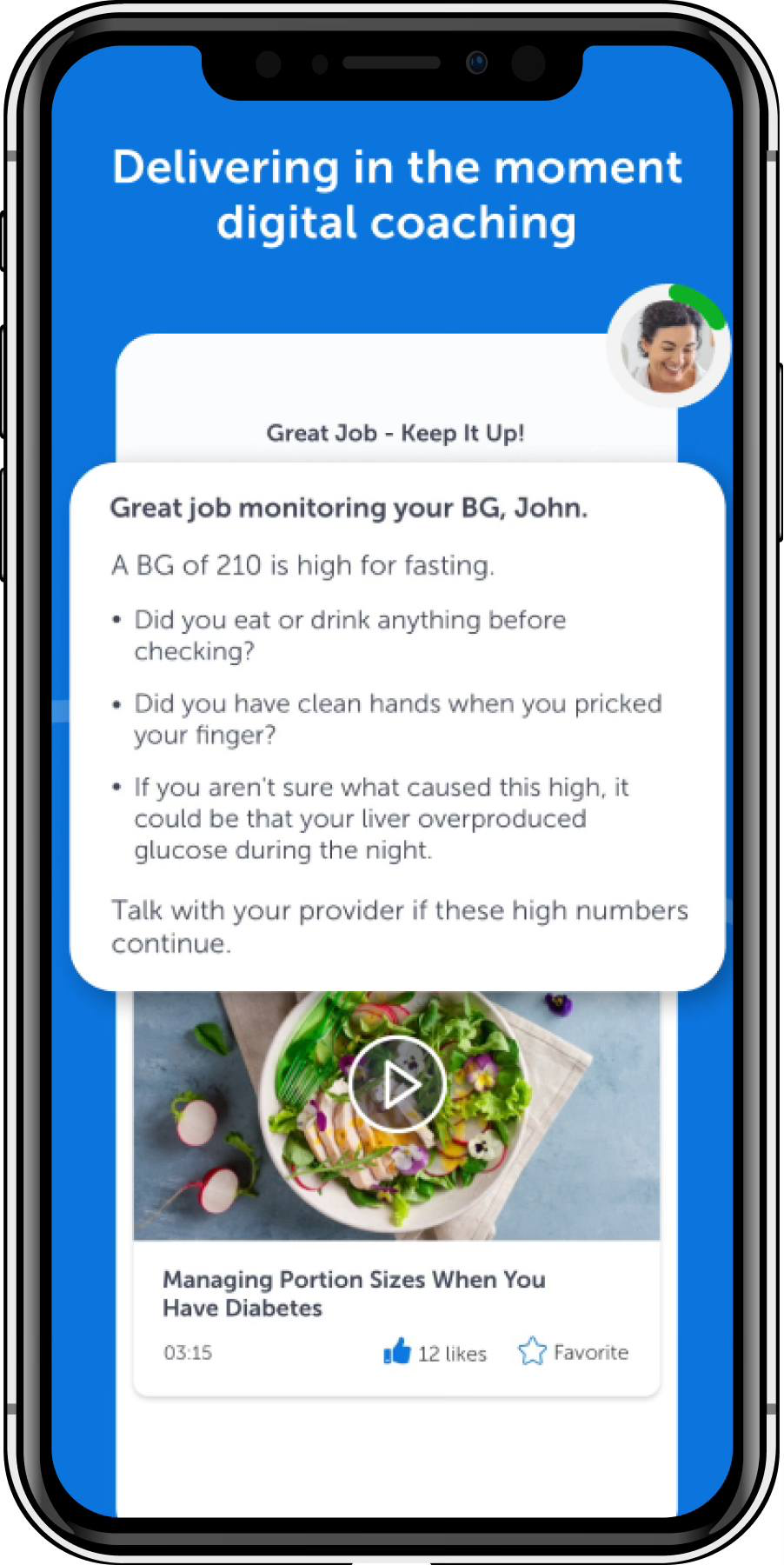

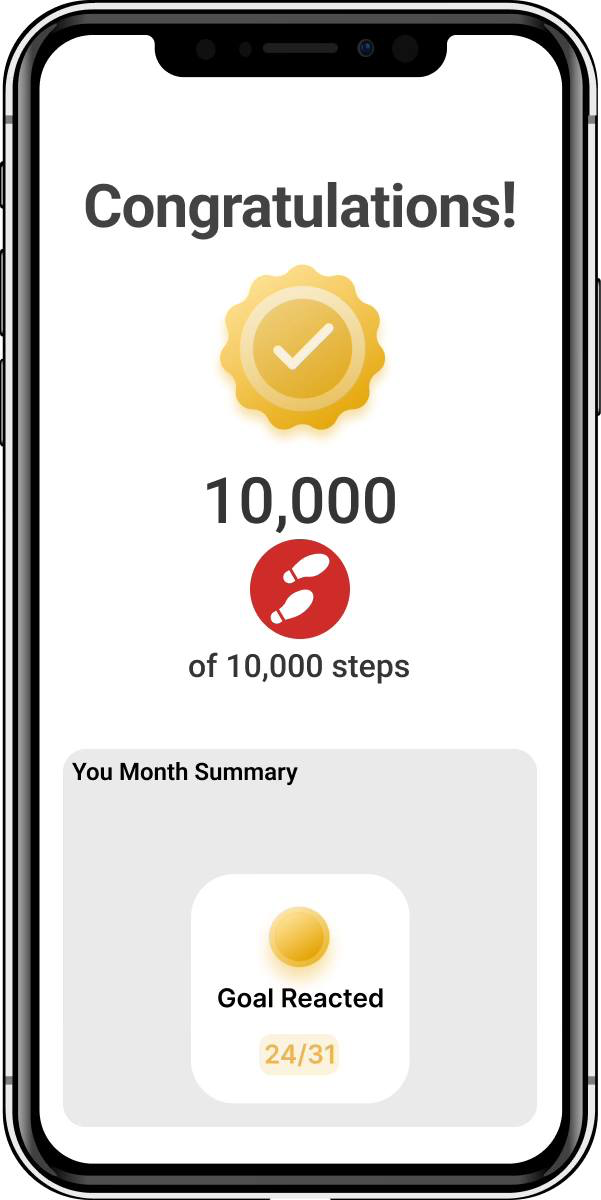

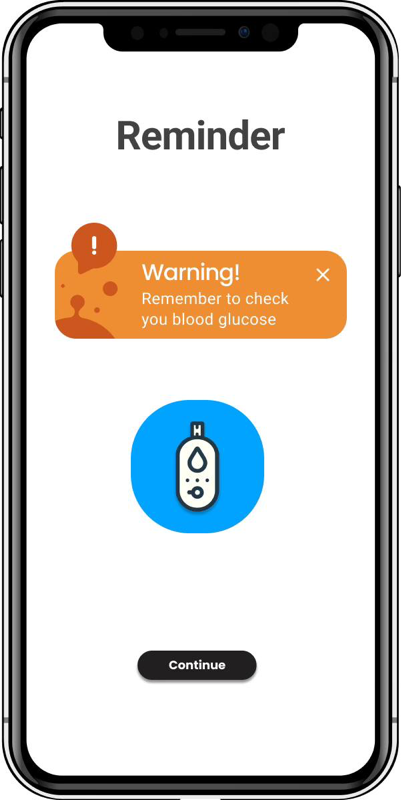

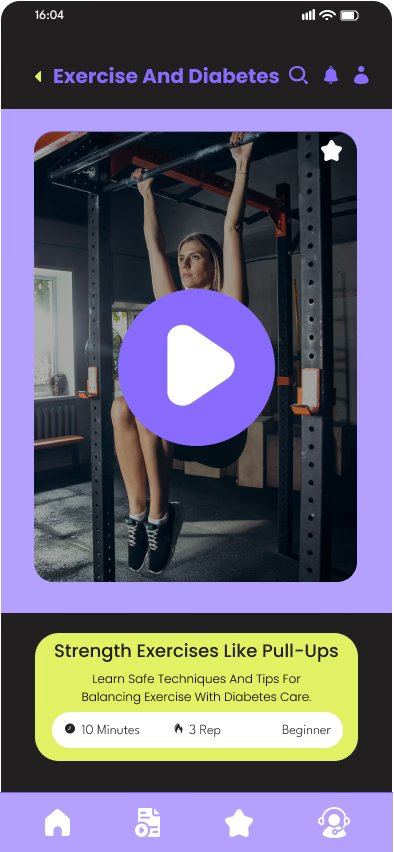

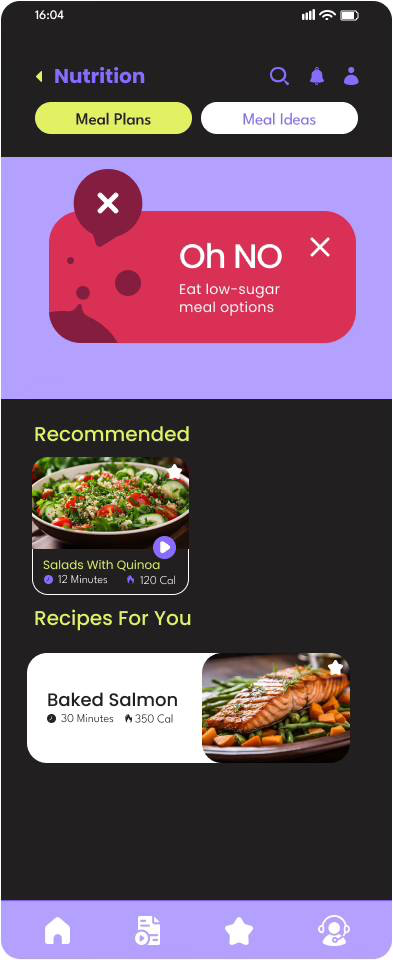

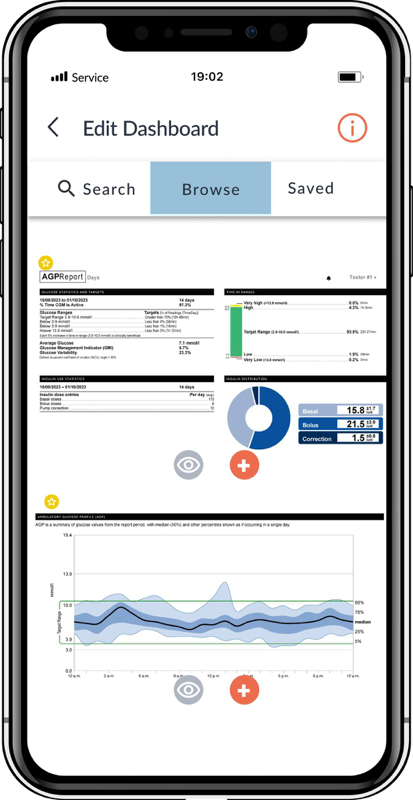

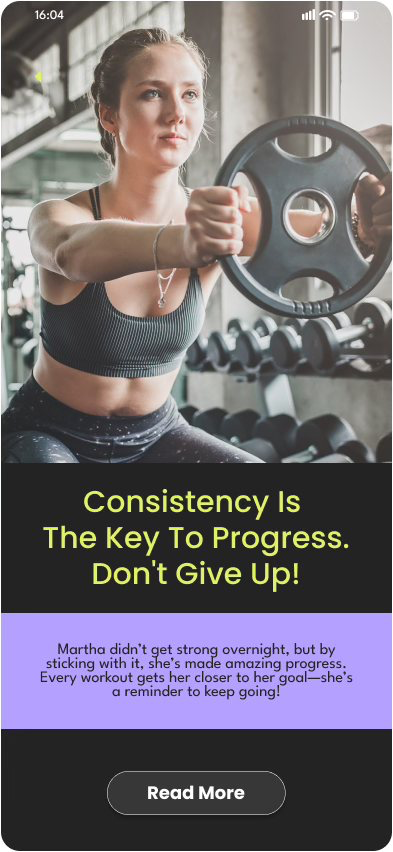

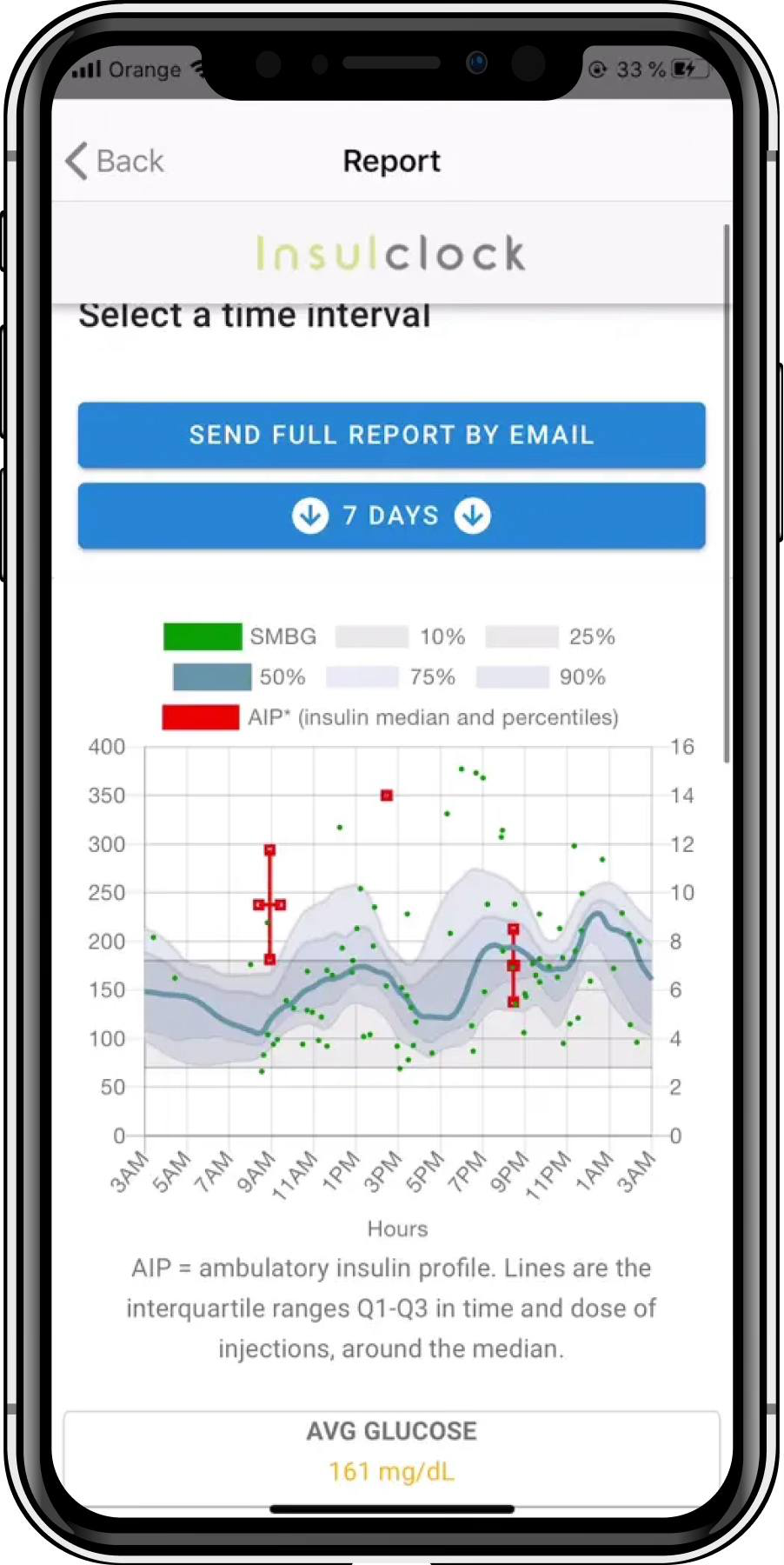

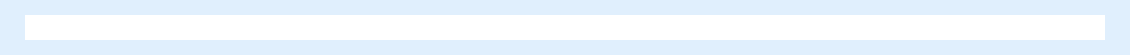

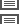

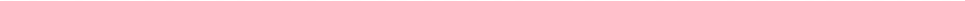

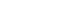


The Importance of User Involvement in Developing Behavioral Apps for Diabetes Management

Page 1

# Making tomorrow's apps more motivational, useful, and effective

Thank you for participating in this questionnaire. Please note the following:

1. **Participation is voluntary**, and **you can withdraw at any time** without any consequences.
2. The data collected (e.g., age and type of diabetes) **is anonymized and cannot be linked to you personally**.
3. By continuing with this questionnaire, **you agree to the use of your anonymized responses for the purposes of this study, i.e. making tomorrow's apps more motivational, useful, and effective.**

[If you have any questions or need further information, please contact eirik.arsand@uit.no](mailto:Ifyouhaveanyquestionsorneedfurtherinformation%2Cpleasecontacteirik.arsand@uit.no)

Page break

Page 2

Mandatory fields are marked with an asterisk *

## Age Range *

Please select your age range

18–24

25–34

35–44

45–54

55–64

65–74

75 and older

## Sex *

What is your sex?

Male

Female

Other

Prefer not to say

## Type of Diabetes *

What type of diabetes have you been diagnosed with?

Type 1

Type 2

Gestational diabetes

Not sure

Do not have diabetes

### Please specify

If your type of diabetes was not listed above, please specify here:

# Behavioural strategies in apps

Below, you will find 9 **examples of Behavioural strategies in apps**, and we want you to rate the expressions associated with these as “Not agree”, “Don’t know”, or “Agree”.

**For each of the nine strategies**, there is an example of usage by an app, but this is just ONE of many ways it can be done. Please rate the expression, and not the specific example.

## Education *

Educational content in the app (e.g., articles, tips, or videos about diabetes management) helps me understand my condition and make better health decisions.

*Example of demonstrating "education" component*

Not agree

Don’t know

Agree

## Persuasion *

Motivational messages or notifications from the app encourage me to stay consistent with my diabetes care.

*Example of demonstrating the "Persuasion" component*

Not agree

Don’t know

Agree

**Incentivization** (Creating an expectation of reward) *

Rewards or badges earned in the app for completing health-related tasks (like exercise or tracking blood glucose) motivate me to use the app regularly.

*Example of demonstrating the "Incentivization" component*

Not agree

Don’t know

Agree

### **Coercion** (Creating an expectation of punishment or cost e.g., penalties for smoking in certain areas) *

Reminders about loss of rewards or badges earned in the app for completing health-related tasks (like exercise or tracking blood gluco- se) motivate me to use the app regularly.

*Example of demonstrating the "Coercion" component*

Not agree

Don’t know

Agree

## Training *

Training resources like video tutorials or step-by-step instructions **boost my confidence** in using apps and following good routines.

*Example of demonstrating the "Training" component*

Not agree

Don’t know

Agree

### **Restriction** (Reducing the opportunity to engage in unwanted behaviours) *

The app’s personalized recommendations (e.g., avoiding or limiting certain foods) help me to manage my diabetes.

*Example of demonstrating the "Restriction" component*

Not agree

Don’t know

Agree

**Environmental Restructuring** (Changing the physical or social environment) *

The app’s ability to customize my dashboard with easy access to essential features (like glucose tracking and meal logging) makes it easier to manage my diabetes.

*Example of demonstrating the "Environmental Restructuring" component*

Not agree

Don’t know

Agree

### **Modelling** (Providing an example for people to aspire to or imitate (e.g., using role models to demonstrate healthy behaviors) *

Seeing examples or success stories in the app of others inspires me to stick with my own goal.

*Example of demonstrating the "Modelling" component*

Not agree

Don’t know

Agree

## Enablement *

I find visual reports, such as charts and graphs, helpful for understanding my health.

*Example of demonstrating the "Enablement" component*

Not agree

Don’t know

Agree
