# Supplementary material for: User Perceptions of Behavioral Change Strategies in Diabetes Apps: Feedback From Online Support Groups
Source: J Diabetes Sci Technol. 2025 May 24;19(5):1239–46. doi: 10.1177/19322968251343918 (PMC12104217; doi:10.1177/19322968251343918)
Supplement: sj-docx-2-dst-10.1177_19322968251343918 – Supplemental material for User Perceptions of Behavioral Change Strategies in Diabetes Apps: Feedback From Online Support Groups [file sj-docx-2-dst-10.1177_19322968251343918.docx]

Spørreskjema om morgendagens diabetesapper


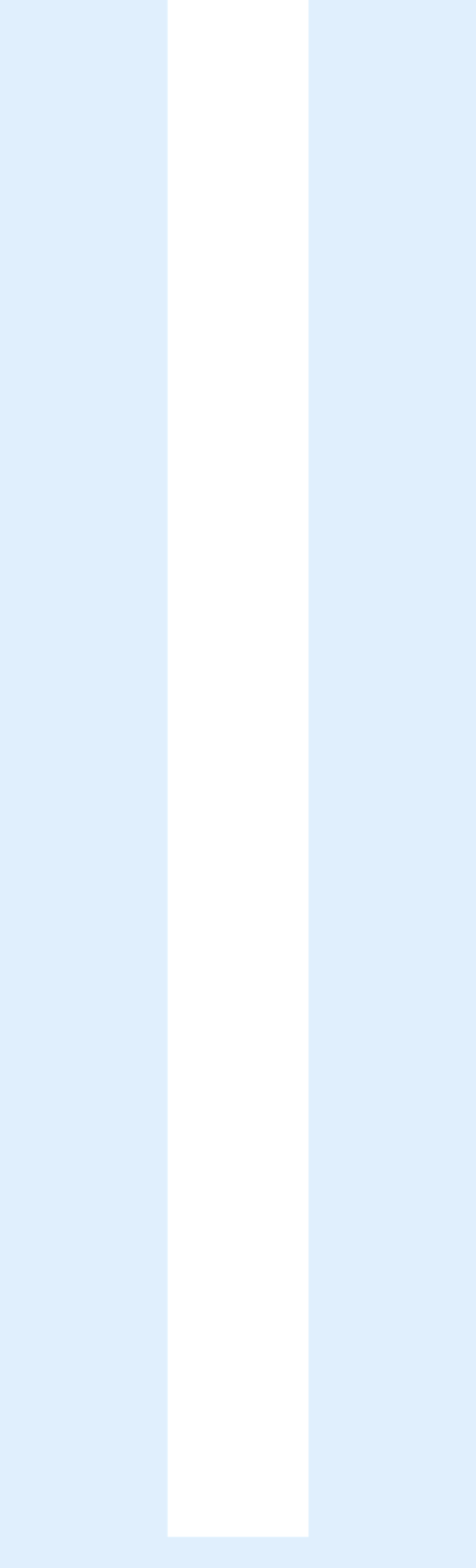

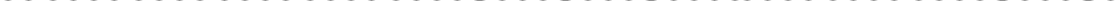

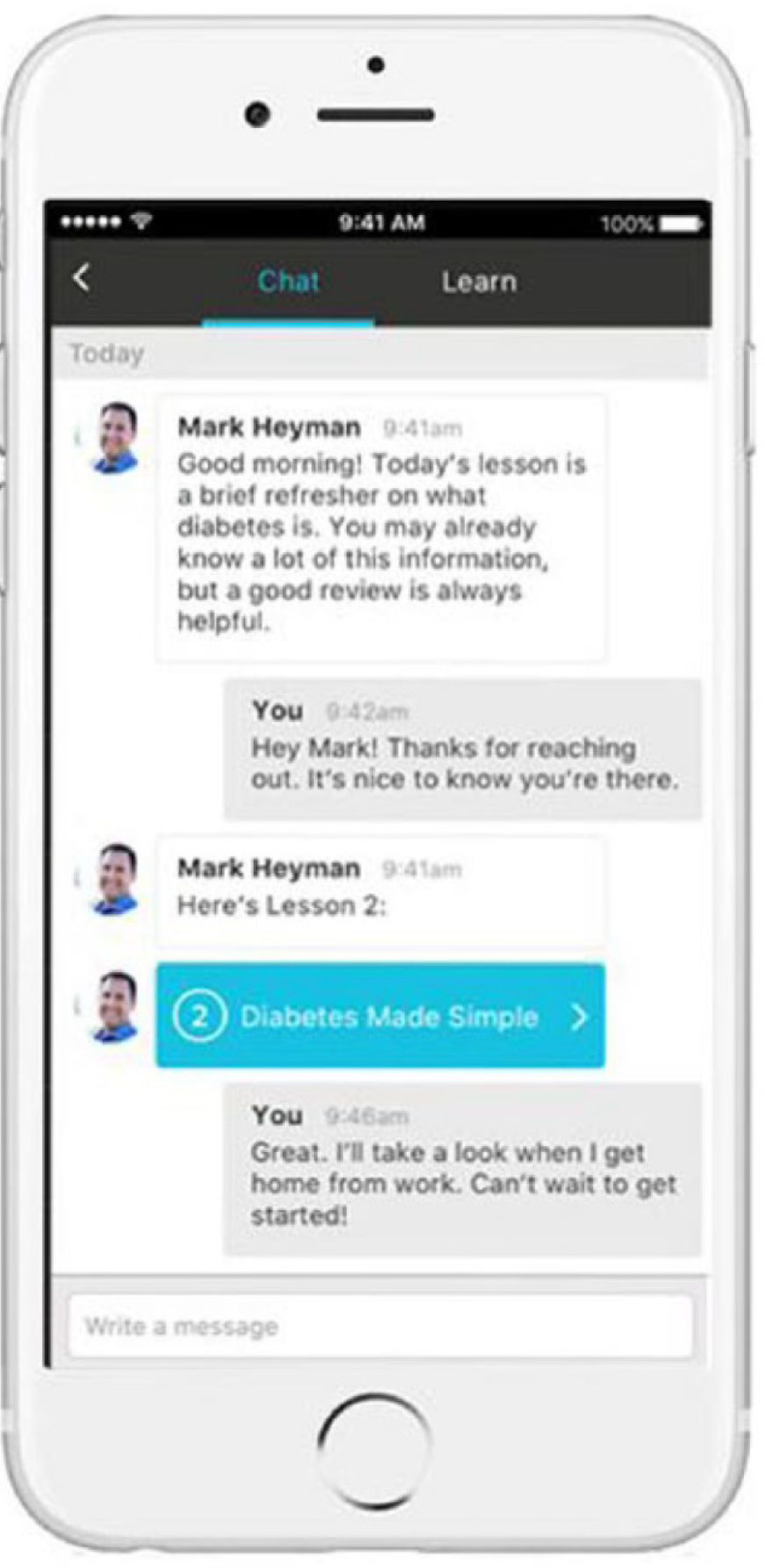

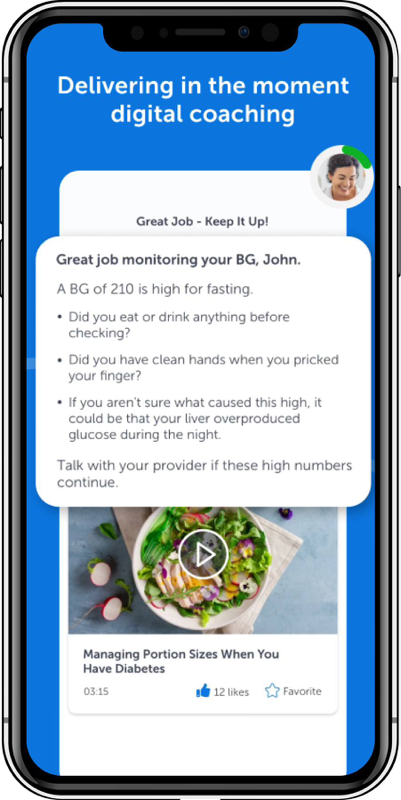

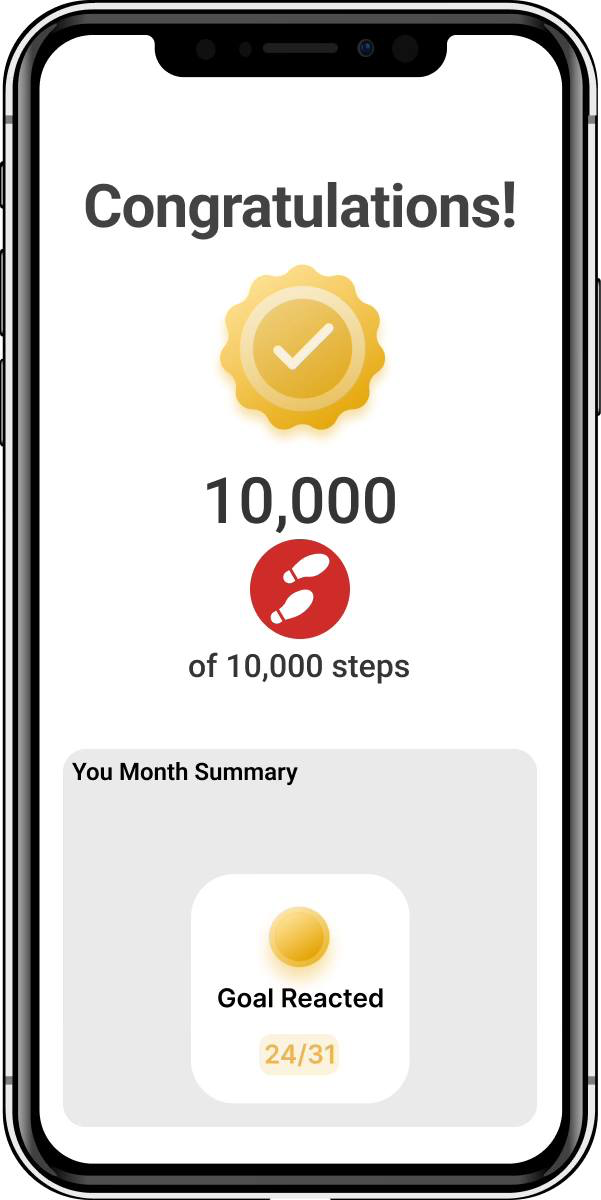

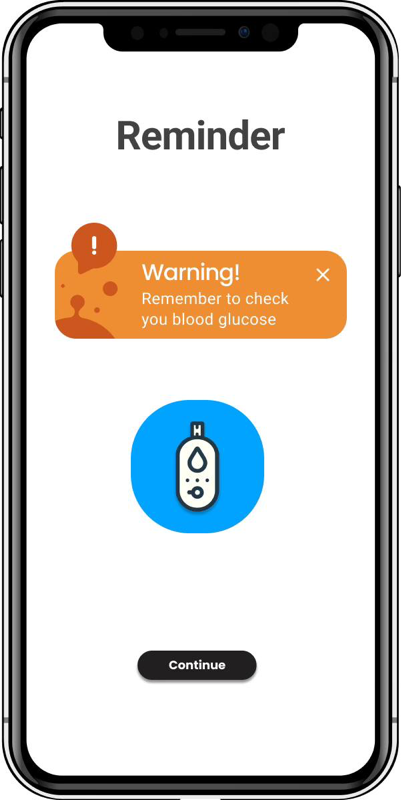

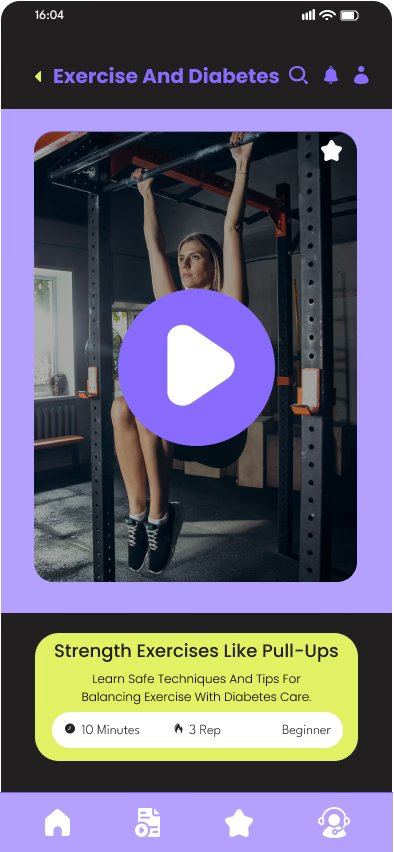

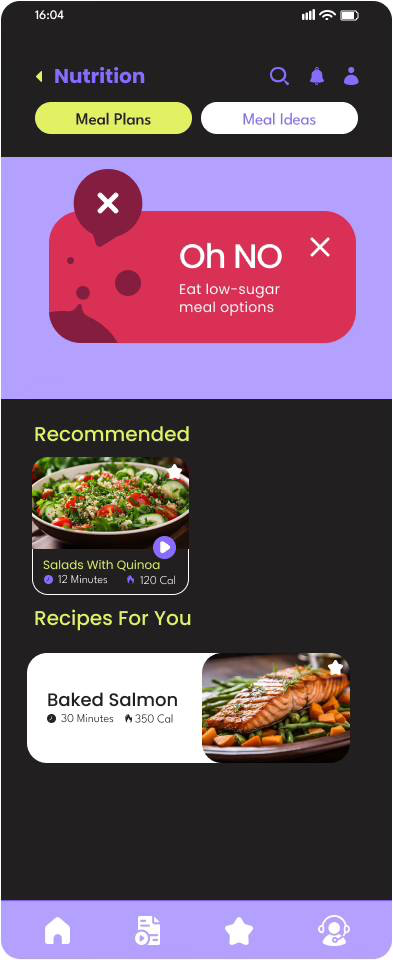

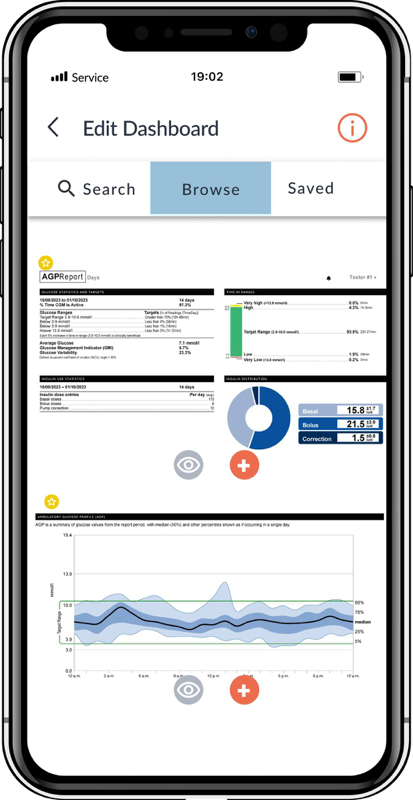

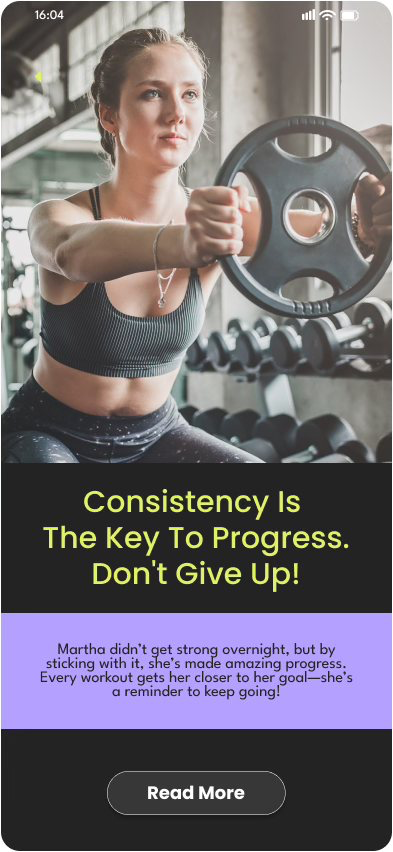

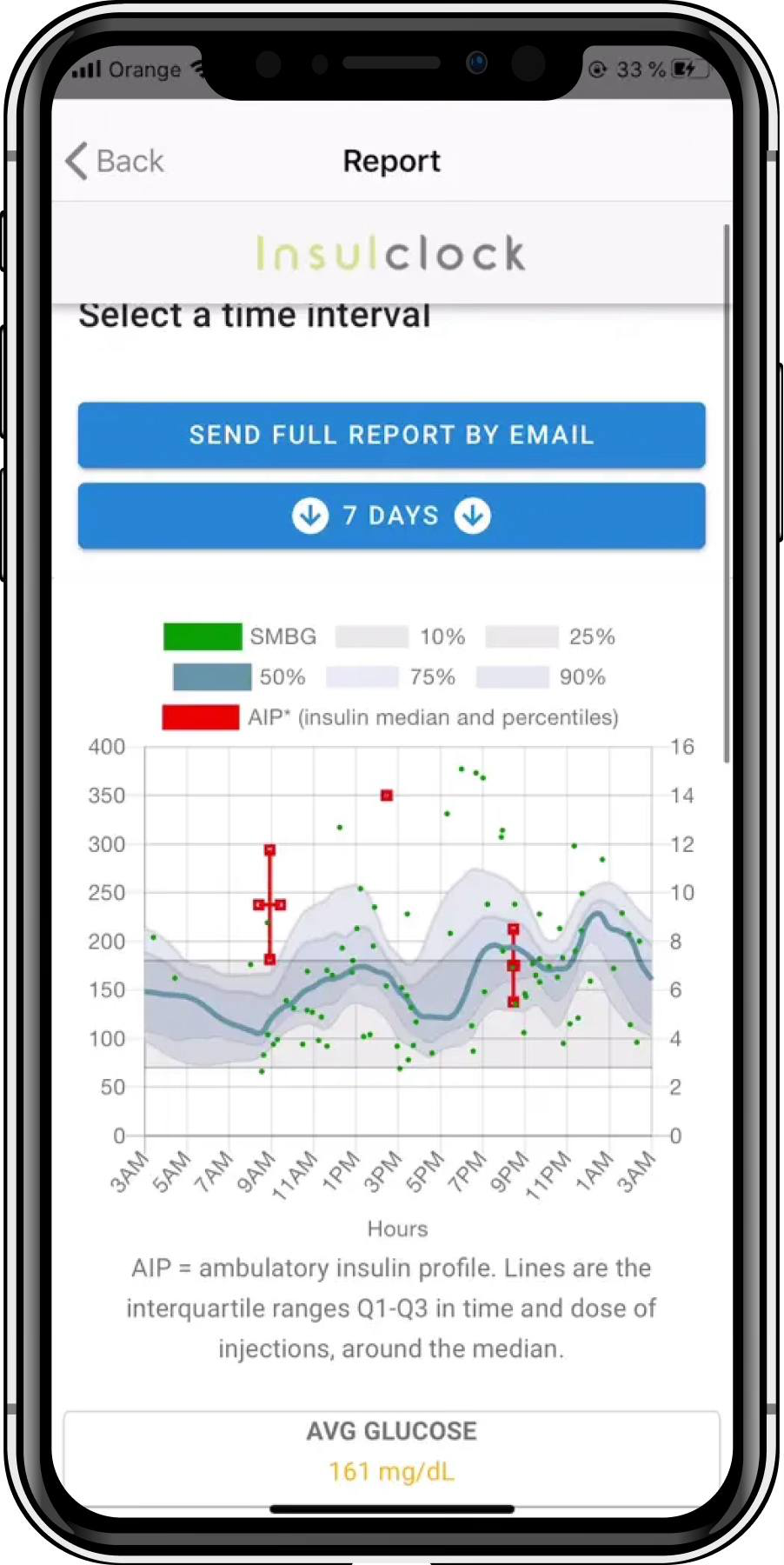

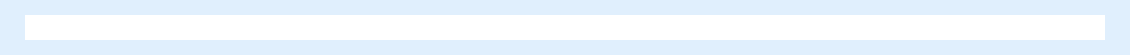

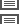

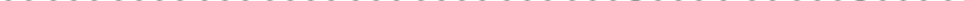

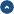

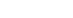


Page 1

# Hvordan gjøre apper mer motiverende, nyttige og effektive

Takk for at du deltar i dette spørreskjemaet. Vennligst merk følgende:

1. Deltakelse er frivillig, og du kan trekke deg når som helst uten konsekvenser.
2. Dataene som samles inn (f.eks. alder og type diabetes) er anonymisert og kan ikke knyttes til deg personlig.
3. Ved å fortsette med dette spørreskjemaet, samtykker du til at dine anonymiserte svar brukes til formålet med denne studien, nemlig å gjøre morgendagens apper mer motiverende, nyttige og effektive.

[Hvis du har spørsmål eller trenger mer informasjon, vennligst kontakt eirik.arsand@uit.no.](mailto:Hvisduharspørsmålellertrengermerinformasjon%2Cvennligstkontakteirik.arsand@uit.no)

Page break

Page 2

Mandatory fields are marked with an asterisk *

## Aldersgruppe *

Vennligst velg din aldersgruppe

18–24

25–34

35–44

45–54

55–64

65–74

75 år og eldre

## Kjønn *

Hva er ditt kjønn?

Mann

Kvinne

Annet

Ønsker ikke å svare

## Type diabetes *

Hvilken type diabetes har du blitt diagnostisert med?

Type 1

Type 2

Svangerskapsdiabetes

Usikker

Har ikke diabetes

### Vennligst spesifiser

Hvis din type diabetes ikke er oppført ovenfor, kan du spesifiser den her:

# Atferdsstrategier i apper

Nedenfor finner du 9 eksempler på atferdsstrategier i apper, og vi ønsker at du vurderer beskrivelsen knyttet til disse som "Ikke enig", "Vet ikke" eller "Enig".

For hver av de ni strategiene er det et eksempel på bruk av disse i en app, men dette er bare ÉN av mange måter det kan gjøres på. Derfor, vennligst vurder beskrivelsen, og ikke det spesifikke eksempelet.

## Utdanning *

Opplæringsinnhold i appen (f.eks. artikler, tips eller videoer om diabetesbehandling) kan hjelpe meg med å forstå tilstanden min og ta bedre beslutninger vedrørende min helse.

*Eksempel på hvordan "utdannings"-komponenten kan se ut:*

Ikke enig

Vet ikke

Enig

## Overtalelse *

Motiverende meldinger eller varsler fra appen kan oppmuntre meg til å være konsekvent med diabetesbehandlingen min.

*Eksempel på hvordan "overtalelses"-komponenten kan se ut:*

Ikke enig

Vet ikke

Enig

**Motivasjon** (Skape en forventning om belønning) *

Belønninger eller merker opptjent i appen for å fullføre helse-relaterte oppgaver (f.eks. trening, blodsukkerkontroll. osv.) kan motivere meg til å bruke appen regelmessig.

*Eksempel på hvordan motivasjons-komponenten kan se ut:*

Ikke enig

Vet ikke

Enig

### **Tvang** (Skape en forventning om straff eller kostnad) *

Påminnelser om tap av belønninger eller merker opptjent i appen for å fullføre helse-relaterte oppgaver (som trening eller blodsukker- kontroll) kan motivere meg til å bruke appen regelmessig.

*Eksempel på hvordan "tvang"-komponenten kan se ut:*

Ikke enig

Vet ikke

Enig

## Opplæring *

Opplæringsressurser som videoopplæring eller trinn-for-trinn-instruksjoner kan øke min selvtillit i å bruke apper og følge gode rutiner.

*Eksempel på hvordan "opplærings"-komponenten kan se ut:*

Ikke enig

Vet ikke

Enig

**Restriksjon** (Redusere muligheten til uønsket atferd) *

Appens personlige anbefalinger (f.eks. til å unngå eller begrense visse matvarer) kan hjelpe meg med å håndtere diabetesen min.

*Eksempel på hvordan "restriksjons"-komponenten kan se ut:*

Ikke enig

Vet ikke

Enig

**Miljøomstrukturering** (Endre det fysiske eller sosiale miljøet) *

Appens evne til å tilpasse utseende, med enkel tilgang til essensielle funksjoner (f.eks. blodsukkerkontroll og måltidsloggføring) kan gjøre det lettere å håndtere diabetesen min.

*Eksempel på hvordan "miljøomstrukturerings"-komponenten kan se ut:*

Ikke enig

Vet ikke

Enig

### **Modellering** (Få deg til å strekke deg etter, eller etterligne rollemodeller, f.eks. noen som har sunne vaner) *

Å se eksempler eller suksesshistorier i appen fra andre kan inspirere meg til å holde meg til mine egne mål.

*Eksempel på hvordan "modellerings"-komponenten kan se ut:*

Ikke enig

Vet ikke

Enig

## Muliggjøring *

Jeg tror visuelle rapporter, som diagrammer og grafer, er nyttige for å bedre forstå helsa min.

*Eksempel på hvordan "muliggjørings"-komponenten kan se ut:*

Ikke enig

Vet ikke

Enig
